# High-Level Expression of Recombinant Bovine Lactoferrin in *Pichia pastoris* with Antimicrobial Activity

**DOI:** 10.3390/ijms17060902

**Published:** 2016-06-09

**Authors:** Blanca Iglesias-Figueroa, Norberto Valdiviezo-Godina, Tania Siqueiros-Cendón, Sugey Sinagawa-García, Sigifredo Arévalo-Gallegos, Quintín Rascón-Cruz

**Affiliations:** 1 Laboratorio de Biotecnología 1, Facultad de Ciencias Químicas, Universidad Autónoma de Chihuahua, Circuito 1, Nuevo Campus Universitario, Chihuahua CP 31125, Mexico; blancaflor_igf@hotmail.com (B.I.-F.); nvaldiviezogodina@gmail.com (N.V.-G.); tsiqueiros@uach.mx (T.S.-C.); sareval@uach.mx (S.A.-G.); 2 Laboratorio de Biotecnología, Campus de Ciencias Agropecuarias, Universidad Autónoma de Nuevo León, Francisco Villa S/N Col. Ex hacienda El Canadá, General Escobedo, Nuevo León 66054, Mexico; ssinagawa@gmail.com

**Keywords:** recombinant bovine lactoferrin, lactoferricin, antibacterial activity, *Pichia pastoris*

## Abstract

In this study, bovine lactoferrin (bLf), an iron-binding glycoprotein considered an important nutraceutical protein because of its several properties, was expressed in *Pichia pastoris* KM71-H under AOX1 promoter control, using pJ902 as the recombinant plasmid. Dot blotting analysis revealed the expression of recombinant bovine lactoferrin (rbLf) in *Pichia pastoris*. After Bach fermentation and purification by molecular exclusion, we obtained an expression yield of 3.5 g/L of rbLf. rbLf and predominantly pepsin-digested rbLf (rbLfcin) demonstrated antibacterial activity against *Escherichia coli* (*E. coli*) BL21DE3, *Staphylococcus aureus* (*S. aureus*) FRI137, and, in a smaller percentage, *Pseudomonas aeruginosa* (*Ps. Aeruginosa*) ATCC 27833. The successful expression and characterization of functional rbLf expressed in *Pichia pastoris* opens a prospect for the development of natural antimicrobial agents produced recombinantly.

## 1. Introduction

Milk is a fluid produced by females of all mammal species whose main function is to be the first food of a newborn because it supplies all the nutritional requirements of the newborn to contribute to organ development and the maintenance of normal physiology [1,2].

Lactoferrin (Lf) was first identified in milk in 1939 by Sorensen [3] and the first reports reference it as “red protein from milk” [4]. It is an 80-kDa iron-binding glycoprotein belonging to the transferrin family. It is found in high concentrations in colostrum and milk, and in lower amounts in mucosal secretions such as tears, saliva, semen, nasal and bronchial secretions, bile, and gastrointestinal fluids [5]. On the other hand, lactoferrin is also a constitutive component of neutrophils; it is released from them in the inflammatory process. Therefore, its concentration is elevated in inflammatory, neurodegenerative, and inflammatory diseases, such as arthritis and the allergic process. Lf is known because of its ability to modulate the immune response. Currently, the mechanism to exert its immunomodulatory activity is not well elucidated; however, reports suggest that Lf is able to recruit several immune cells, as well as promote the balance of the production of these cells [6]. Other important functions of Lf are related to its antimicrobial effects which have been demonstrated *in vivo* and *in vitro* [7]. Lf is capable of affecting the proliferation of several microorganisms with clinical importance, including Gram-positive and -negative bacteria, and viruses such as HIV, parasites, and fungi [8,9]. The antibacterial effect of Lf has been noticeably more studied; bioactive peptides of Lf have the ability to bind to some molecules of the cell membrane, causing instability and subsequent cell disruption. Moreover, its capacity to sequester iron allows competition for nutrients with siderophores, avoiding their growth and proliferation [10,11]. Other qualities render Lf a multifunctional protein, such as its anticarcinogenic and enzymatic activity [5].

Lf has been named “nutraceutical protein” because of its remarkable potential to have multiple properties and the potential for use as a therapeutic protein [12]; it is considered one of the most produced recombinantly [13,14]. Lf has been expressed in several microscopic models. Our research group has expressed bovine lactoferrin (bLf) using an *Escherichia coli* (*E. coli*) model expression system, and we obtained functional antibacterial activity fractions of bLf [15]; however, the use of prokaryotic systems presents disadvantages which can be avoided by using eukaryotic systems—for example, the protein toxicity could interfere with growth and cause cell death [16].

In the pharmaceutical industry, yeast-based expression systems are highly valued. In recent years, the production of recombinant proteins has increasingly focused on the yeast. *Pichia pastoris* (*P. pastoris*) is methylotrophic yeast belonging to the Ascomycetes group. It is characterized by its ability to have a strong regulator promoter, its rapid growth, and its easy genetic manipulation; moreover, it has the ability to perform eukaryotic post-translational modifications. *P. pastoris* characteristics have made successful use possible for the production of many recombinant proteins, several of which are drugs currently in clinical development [17,18].

Several kinds of Lf have been expressed in *P. pastoris*. Lf from humans, horses, pigs, goats, and sheep has been expressed and purified. The expression levels obtained range from 2 to 1200 mg/mL, and lactoferricin and lactoferampin, its bioactive peptide, have also been expressed in *Pichia pastoris* [17,18,19]. However, there are no reports of bLf expression in *P. pastoris*; bLf shares high homology among species, and its antimicrobial potential has been tested *in vivo* and *in vitro* [20].

Due to the growing problem of antibiotic resistance, we focus our research on developing technologies that allow for the obtainment of rLf with a powerful antimicrobial effect, which can be used in the future as a replacement to current antibiotics. In the present study, we report for the first time the expression, purification, and potential antimicrobial activity of recombinant bovine lactoferrin (rbLf) in *Pichia pastoris.*

## 2. Results

### 2.1. Expression Vector Design and Synthesis

**Figure 1:**
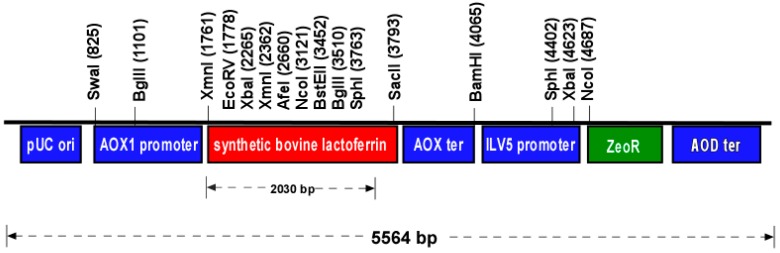
Restriction map of pJ902sbLf. The vector containing a synthetic *bovine lactoferrin* (*sbLf*) gene for expression in *P. pastoris* yeast. The *ZeoR* gene was also included as a selectable marker.

The vector pJ902sbLf (Figure 1) encodes for the bLf with optimized codon usage for *P. pastoris* expression; it contains a selectable marker which confers Zeocin resistance. The synthetic *bovine lactoferrin* (*sbLf*) gene is derived from a *bLf* gene (GenBank accession No. EU812318) cloned by García-Montoya *et al.* [15] and is under the control of the AOX1 promoter. Alkaline lysis extraction and digestion of the synthetic plasmid pJ902sbLf by BamHI produced a linearized DNA fragment that measured 5564 bp in length. 

### 2.2. Molecular Analysis of Peptides Derived from Lactoferrin

**Figure 2:**
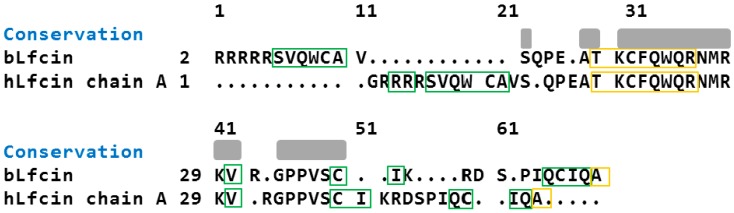
Comparison of bovine and human Lfcin moiety. Upper gray box represents amino acid identity. Green boxes represents beta sheet and yellow box represent alfa sheet structure homology.

An alignment analysis of the human and bovine LfcinAB was done (Figure 2) using a protein alignment tool (UCFs Chimera, San Francisco, CA, USA). The overall amino-acid-based structure identity was 43.7%.

**Figure 3:**
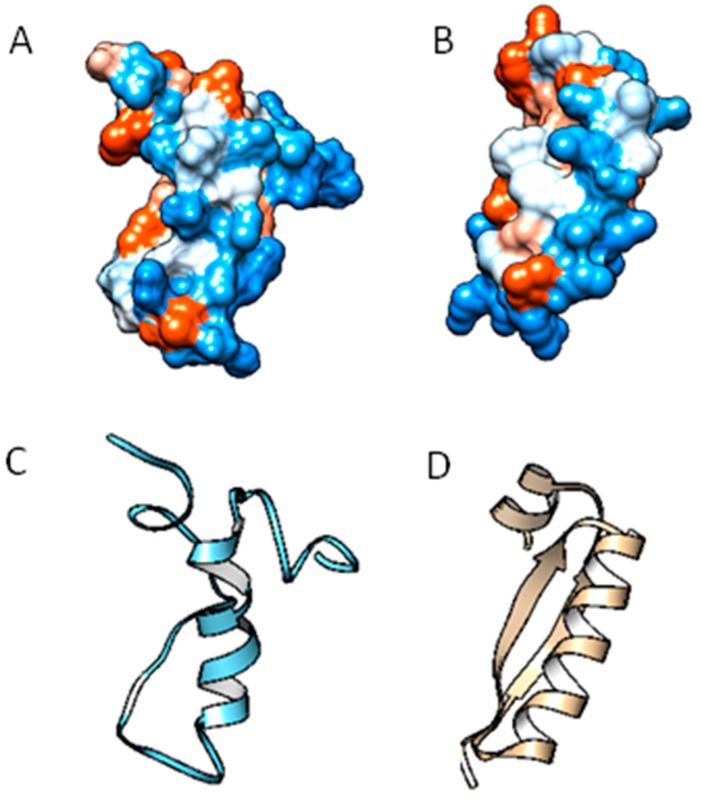
Tertiary model structure of human and bovine Lfcin. (**A**) Hydrophobicity model hLfcin domain (residues 17–41); PDB ID: 1BLF; and (**B**) bovine Lfcin (residues 17–31) modeled with 1bLf as template; (**C**) predicted tertiary model of hLfcin and (**D**) bLfcin. Viewed using Chimera software (UCFs Chimera, San Francisco, CA, USA).

LFcin identification can be made on the basis of critically conserved basic residues—Lys in positions 35, 41; Trp in 28; and Arginine in 37 and 40—represented by light-blue-colored letters in the hydrophobicity modeling of Lfcin in Figure 3A,B. All have important roles in the interactions with negatively charged surfaces, such as the lipopolysaccharide (LPS)-containing outer membrane (OM) of Gram-negative bacteria (*E. coli* and *Pseudomonas aeruginosa* (*Ps. aeruginosa*)) and the teichoic acid layer that surrounds the cytoplasmic membrane of Gram-positive bacteria (*Staphylococcus aureus* (*S. aureus*)).

The tertiary structure of Lfcin is markedly different (Figure 3C,D); however, a conserved central α-helix is observed in the Lf structure.

### 2.3. Expression and Purification of Recombinant Bovine Lactoferrin (rbLf)

**Figure 4:**
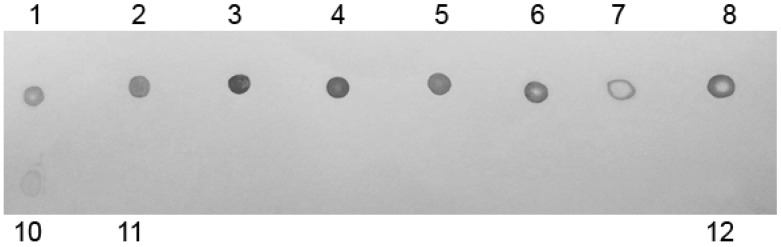
Dot-immunoblotting analyses of recombinant bovine lactoferrin (rbLf) from induced clones. Following 72 h of induction, proteins were harvested from supernatant and were spotted in a polyvinylidene difluoride (PVDF) membrane. Lanes 1–8: protein samples of each clone; Lane 10: commercial bovine lactoferrin (bLf) (0.5 mg/mL); Lane 11: commercial bLf (0.05 mg/mL); Lane 12: protein extracts from native *P. pastoris*.

**Figure 5:**
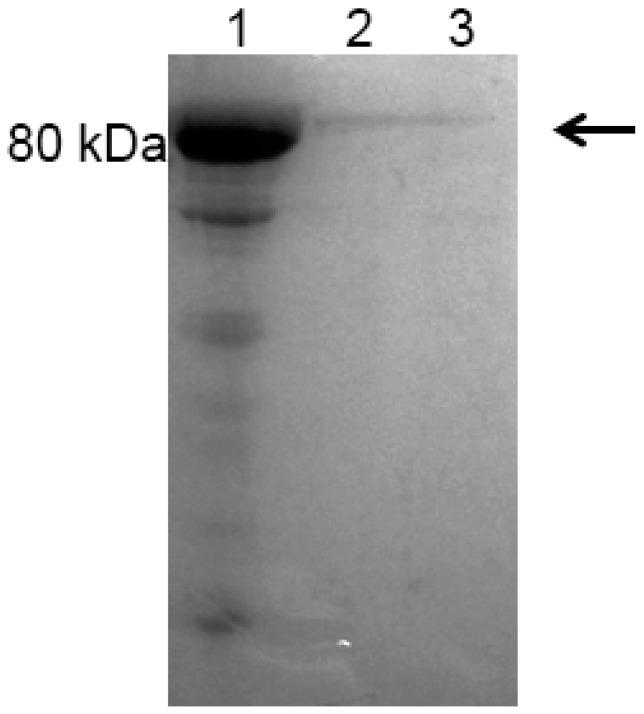
Sodium dodecyl sulfate polyacrylamide gel electrophoresis (SDS-PAGE) analyses of rbLf purified from the yeast culture. Lane 1: commercial bLf; Lanes 2–3: purified rbLf.

After *P. pastoris* transformation with pJ902sbLf vector, eight transformant clones were obtained. Protein extracts were recovered, and dot blotting analyses after 72 h of induction revealed the presence of lactoferrin in all clones; rbLf was detectable because of a very low concentration (0.5 mg/mL), according to the detection of commercial bLf concentrations (Figure 4). The best-producing clone was cultured in a batch fermentation using a shake flask, and the total protein extracts were recovered and purified. Sodium dodecyl sulfate polyacrylamide gel electrophoresis (SDS-PAGE) confirmed the presence of a band of about 80 kDa, according to lactoferrin weight (Figure 5). After the recombinant protein induction on a large scale, we increased the expression level and obtained 3.5 g/L of rbLf.

### 2.4. Antibacterial Activity of rbLf

**Figure 6:**
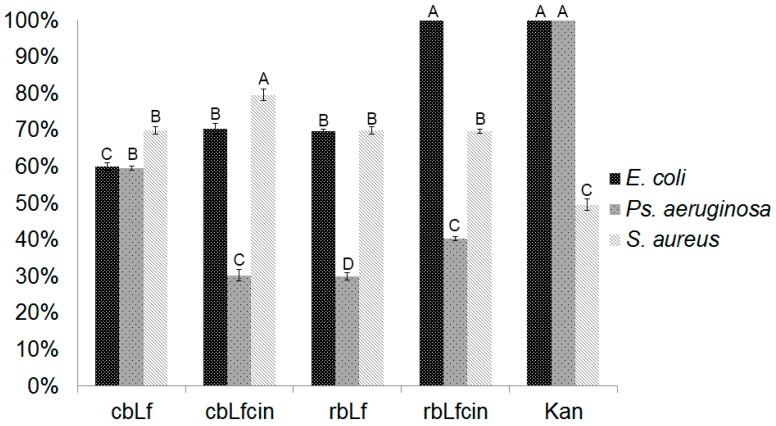
Antibacterial activity of rbLf. Bacterium were incubated in Luria-Bertani (LB) broth and treated with 5.0 ng/mL. Inhibition growth percentage was calculated relative to commercial bLf and commercial antibiotics. cbLf = commercial bovine lactoferrin; cbLfcin = commercial bovine lactoferricin; rbLf = recombinant bovine lactoferrin. rbLfcin = recombinant bovine lactoferricin; Kan = kanamycin. Group A belongs to treatment with highest percentage of inhibition, while group D belongs to treatment with lowest inhibition. Groups B and C are intermediate, however they are statistically different.

The antibacterial potential of rbLf was confirmed using non-fragmented and pepsin-digested rbLf. After incubation in a 96-well microplate, we established a bacterial growing inhibition percent based on a Rezarsurine indicator, which presents a blue coloration in the absence of bacterial growth. In the presence of a bacterial growth, it veers towards a pink color as a consequence of a REDOX reaction because of the bacterial metabolism. According to the different observed hues in the presence of Rezarsurine in the 96-well microplate, we set arbitrary values of bacterial growth inhibition percent based on the pink hue. This data indicated a percentage of bacterial growth of 0%; the blue hue indicated that the percentage of bacterial growth inhibition was 100%. Percentages were assigned by reference to the visual color scale PANTONE^®^ (Pantone Inc., Carlstadt, NJ, USA). The highest percentage of inhibition was achieved in the presence of pepsin-digested rbLf by the action of its bioactive peptide lactoferricin. An amount of 5.0 ng/mL was enough to achieve a growth inhibitory effect. *E. coli* was totally inhibited using rbLfcin, while the strain *S. aureus* was able to inhibit 70% only. For *Ps. aeruginosa*, a 40% inhibition using rbLfcin was obtained (Figure 6). One-way ANOVA showed that the difference between the use of rbLfcin (Group A) and the use of antibiotic kanamycin (Group A) to inhibit *E. coli* growth was not significant. rbLf (Group B) presented a significant difference about rbLfcin. In the treatment against *Ps. aeruginosa* growth, a significant difference was observed between kanamycin (Group A), rbLfcin (Group C), and rbLf (Group D)*.* Finally, when rbLf was tested to inhibit *S. aureus* growth, a major inhibition percentage using cbLfcin (Group A) was obtained; rbLf and rbLfcin (Group B) showed a smaller inhibition percentage than cbLfcin; however, a lower percentage was obtained with the use of antibiotic kanamycin (Group C). These results indicated that the best way to use rbLf against microorganisms is in its digested form, rbLfcin, and the most susceptible bacterium is *E. coli*.

## 3. Discussion

Because of the indiscriminate use of antibiotics, the incidence of infections caused by resistant strains is currently rising. The development of new drugs has slowed, so the search and generation of new treatment strategies become an essential alternative [21]. Moreover, bLf has been used successfully to prevent and treat infectious diseases in newborns. When bLf is added to infant formulas, the incidence of infectious diseases of the respiratory tract and diarrhea decreases [22]. On the other hand, the oral administration of bLf generates a protective effect against necrotizing enterocolitis in very-low-birth-weight neonates [23]. These reports suggest that, in the future, rbLf could be used safely in neonates and will have a positive impact on their development and health. bLf displays potential antimicrobial activity, included against resistance strains. We produce and evaluate rbLf expressed in *Pichia pastoris* yeast and its antimicrobial effects.

The methylotrophic yeast *Pichia pastoris* has been one of the mostly used production platforms of recombinant proteins of pharmaceutical interest for over three decades [24]. The use of *Pichia pastoris* as an expression system of therapeutic proteins gave us the advantage of combining the easy handling and minimum growth requirements of a unicellular organism with the easily achievable genetic modifications of a eukaryotic system.

An alignment analysis of the Lf-derived peptides was performed (Lfcin A and B chain); despite its differences in nucleotide sequence, amino acid similarity was evident. This region retains high homology among mammals and corresponds to amino acids 12–48 [25]. Chemically synthesized fragments from Lfampin 268–284 and Lfampin 265–284 differ in only three amino acids (265Asp-Leu267-Ile268), but its antimicrobial activity behavior is different [26]. Such structure may be involved in recognizing bacterial membrane topology [27]. A single β-sheet strand in bovine Lficin has not been observed in human Lfcin structure.

To express rbLf in *Pichia pastoris* KM71-H, we used the *sbLf* gene, which was modified to optimize the codon usage. Expression of *rbLf* was under the control of the AOX1 promoter. BamHI site was included at the end of the AOX1 terminator region for gene integration. To increase expression levels, a batch fermentation was carried out; the highest expression levels of recombinant proteins under the control of the AOX1 promoter have reached 22 g/L of intracellular proteins [28]. With the feature of being a very strong promoter and being easily controllable by the change in the carbon source, the AOX1 promoter is the most used regulatory element in the expression of heterologous proteins in *P. pastoris*. After 72 h of induction, cell lysis, and purification from 1 L of culture medium, we obtained an expression yield of 3500 mg/L of rbLf. In terms of yield, our expression level has been the highest level of expression of rLf in *Pichia pastoris*. In fact, it places us above of the highest value reported, corresponding to 1200 mg/L [29]; however, this level of expression was obtained from approximately 3 L of culture medium, three times more than that from the present experiment. Other contributions have reported an rLf expression of 60 mg/L from Tibetan sheep [30], 12 mg/L from pigs [31], 40 mg/L from horses [32], 40 mg/L from yaks [33], and a smaller level of expression of 2 mg/L from goat lactoferrin [34].

Accumulation levels of heterologous proteins in yeast are altered by a variety of genetic and physiological factors: the codon usage, the gene copy number, and the kind of promoter, translation signals, and the final destiny of proteins. Secretion into the medium requires necessarily the use of a signal sequence on the foreign protein to target it to the secretory pathway [35]. The use of a secretion signal peptide has the advantage of excreting recombinant proteins to culture media without the need for cell lysis; however, intracellular proteins are accumulated earlier than secreted proteins, so the heterologous proteins can localize in a cytosolic soluble fraction and in an insoluble part protected by inclusion bodies [36]. Surprisingly, our expression levels for rbLf were obtained without the addition of signal sequence, unlike the other works cited above. Heterologous proteins derived from insoluble fractions were released from inclusion bodies with urea, and they actually showed less degradation than the proteins obtained from the soluble fraction.

Finally, we evaluate the antibacterial activity against Gram-negative bacteria *E. coli* and *Ps. aeruginosa* and the Gram-positive bacteria *S. aureus.* Currently, Gram-negative bacteria are becoming increasingly resistant to common antibiotics due to the presence of an outer membrane (OM) that has a unique structure creating an impermeable layer, which provides protection against antibiotic penetration [37]. In our last paper, we demonstrated that, unlike non-fragmented lactoferrin, pepsin-digested rbLf has a higher antibacterial potential against *E. coli* [15]. The proteolytic digestion of Lf by gastric pepsin releases a bioactive peptide of Lf called lactoferricin; because lactoferricin can alter the permeability of bacterial membrane, several studies support the idea that lactoferricin presents higher antibacterial activity that non-fragmented lactoferrin [38]. We proved that digested rbLf was able to inhibit 100% of growth of *E. coli*. Peptide insertion into the bilayer membrane of *E. coli* generates a void in the hydrophobic core. In response to this void, the bacterium compensates trying to fill it with the monolayer, which generates a thinning of the membrane with a consequent dispersion of its components and finally the rupture [39,40]. Therefore, the use of rbLfcin against this enterobacteria is the best way to inhibit its growth. On the other hand, *Pseudomonas aeruginosa* is characterized to be extremely resistant to antibiotic agents [21]; it is an opportunist pathogen implicated mainly in nosocomial infections, and its intrinsic resistance is attributed to its capability to growth in biofilms. Moreover, the expression of efflux pumps allows the expulsion of antibiotics, making it in a multi-resistant strain [41,42]. Despite the difficulty of inhibiting the growth of this bacterium, surprisingly, our digested rbLf was able to inhibit 40% of growth; nevertheless, cbLf inhibited 60% of bacteria growth. Therefore, these findings open a door to the study of the possible mechanisms of action of Lf against this bacteria, which may be involved in part due to the iron sequester, or the membrane lysis caused by the bioactive peptide lactoferricin. Glycosylation sites present in the rbLf may also have a significant impact on the immunological mechanism and antimicrobial action of lactoferrin. The glycosylation pattern can have differences between native and recombinant proteins; however, more studies are needed to support this theory. In the end, the activity of rbLf against Gram-positive bacteria *S. aureus* resulted in the inhibition of bacterial growth until 70%, both rbLf as rbLfcin. The antimicrobial activity against *S. aureus* is implicated in the iron sequester. rLf has shown the bacteriostatic activity of this bacteria [43]. Moreover, a recent study demonstrated that recombinant lactoferricin can achieve the inhibition of *S. aureus* [17], so the lactoferricin mechanism against Gram-positive bacteria should also be involved in the damage to the bacterial membrane by penetration and consequent lysis.

## 4. Experimental Section

### 4.1. Strains, Enzymes, and Plasmids

*Escherichia coli* (*E. coli*) DH5α (Invitrogen^®^, Carlsbad, CA, USA) was used as a host for plasmid propagation. *Pichia pastoris* (*P. pastoris*) KM71-H (provided by Cinvestav, Irapuato, Mexico) was used for protein expression. We use the plasmid pJ902sbLf containing a synthetic gene which encodes for the *bovine lactoferrin* DNA 2.0 in *P. pastoris* (DNA 2.0, Newark, CA, USA). All restriction enzymes were purchased from Invitrogen^®^ (USA). *E. coli* BL21DE3, *S. aureus* FRI37, and *Ps. aeruginosa* ATCC 27833 (provided by the Microbiology Laboratory at Facultad de Ciencias Químicas Universidad Autónoma de Chihuahua) were used for the antimicrobial assay.

### 4.2. Media and Growth Condition

*E. coli* DH5α was cultured at 37 °C in Luria–Bertani (LB) broth (10 mg/L tryptone, 5 mg/L yeast extract, 10 mg/L NaCl) and LB plates (15 mg/mL agar) supplemented with zeocin (37.5 µg/mL) as the selective marker. *P. pastoris* was cultured at 30 °C and 250 rpm on YPD (1% yeast extract, 2% peptone, 2% dextrose) broth and YPD plates (20 mg/mL agar) supplemented with zeocin (175 mg/L) as the selective marker. *E. coli* BL21DE3, *S. aureus* FRI137, and *Ps. aeruginosa* ATCC 27833 were cultured at 37 °C in LB broth.

### 4.3. Transformation of Pichia pastoris KM71-H

Expression vector pJ902sbLf was propagated in *E. coli* DH5α, and the plasmid was isolated from the positive transformant clones. One microgram of linearized plasmid DNA was used to transform *P. pastoris* KM71-H. The transformation was achieved by electroporation using a MicroPulser BioRad^®^ (BioRad, Hercules, CA, USA) according to the manufacturer’s instructions. After 3 days of incubation in the presence of zeocin, the transformant clones were isolated for analyses.

### 4.4. Expression of rbLf

To carry out the rbLf expression, a single colony of transformant clones were inoculated into 5 mL of YPD medium supplemented with zeocin ( 175 mg/mL) and incubated at 250 rpm and 30 °C for 24 h, and the cells were harvested by centrifugation and incubated at a seeding density of 0.1–0.2 optical density (OD) in BMMY medium (100 mM potassium phosphate, pH 6.0, 1% yeast extract, 2% peptone, 1.3% Yeast Nitrogen Base (YNB), 400 µg/L biotin and 0.5% methanol) to induce the expression of rbLf. Methanol was again added to a final concentration of 0.5% (*v*/*v*) every 24 h for 4 days to maintain the induction according to the method described in [29]. After 4 days, the cells were harvested by centrifugation at 5000 rpm for 10 min at 4 °C, and the pellet was resuspended in lysis buffer (10 mM Tris-HCl, 100 mM NaCl at pH 6.5, 1X Protease Inhibitor Cocktail (cOmplete™, Mini, EDTA-free Roche^®^, Pleasanton, CA, USA). The lysate was centrifuged at 5000 rpm for 5 min at 4 °C to obtain soluble proteins. The insoluble pellet which contained inclusion bodies was washed in lysis buffer with 6 M urea and incubated for 1 h on ice and centrifuged at 5000 rpm for 30 min at 4 °C to obtain insoluble proteins. The soluble and insoluble protein extracts were collected and stored at −20 °C for further study.

### 4.5. Batch Fermentation

To increase the Lf accumulation, batch fermentation was achieved as follows: 200 mL of YPD media supplemented with zeocin (175 mg/L) were inoculated with cells from a single colony of the transformant clones and incubated overnight with shaking. Afterward, this culture was added to the fermentor, which contained 1000 mL of BMMY induction media supplemented with zeocin (175 mg/L). The culture was incubated at 30 °C on shaking. The induction stage was initiated after 24 h of growth by adding 5% methanol each 24 h during 72 h to maintain the optimal expression of rbLf.

### 4.6. Purification of rbLf

After induction, protein extracts were collected as described below and purified by precipitation and molecular exclusion. First crude extracts were precipitated with 28% of ammonium sulfate resuspended and dialyzed against 100 vol of phosphate buffer. Semi-purified extracts were applied to a sephacryl S-200 HR Column (GE Healthcare, Aurora, OH, USA) pre-equilibrated with four column volumes of lysis buffer. Then, the column was washed with wash buffer (20 mM Tris-HCl and 150 mM NaCl at pH 7.9). All of the fractions were collected and applied to a 12% SDS-PAGE analysis.

### 4.7. Dot-Blot Analysis 

To assess Lf accumulation, the protein samples were spotted in a PVDF membrane (Immobilon^TM^-P Millipore, Darmstadt, Germany). The membrane was blocked with 1% Bovine Serum Albumin (BSA) in Phosphate Buffered Saline (PBS) and shaken for 2 h. The rbLf was detected using an anti-bLfHRP polyclonal antibody (United States Biological, Salem, MA, USA). The bound antibody was detected using Diaminobenzidine (DAB)/Peroxide substrate system for color development. The expression level was determined via dot-blot by densitometry in a KODAK^®^ Gel Logic 100 System (KODAK^®^, Rochester, NY, USA).

### 4.8. rbLf-Bioactive Peptide Recovery and Antimicrobial Assay 

To test the antimicrobial activity, rbLf was digested with Pepsine (Invitrogen) to release the bioactive peptides according to the protocol reported by Bellamy (1992) [44]. Approximately 2 × 10^8^ Colony Forming Units (UFC) per mL of each strain were incubated in a 96-well microplate (Corning, Corning, NY, USA) containing LB broth with rbLf and pepsin-hydrolyzed rbLf. The final concentration in each well was 5.0 ng/L of rbLf and pepsin-hydrolyzed rbLf. A portion of 0.01% resazurin was added to each well as a REDOX indicator of the cell viability. Untreated strains cultured in LB were used as a control. Commercial bLf (5 ng/mL) and kanamycin (60 ng/mL) were used as a control of growth inhibition. Cultures were incubated at 37 °C for 1 h and monitored by color change as a consequence of the oxidative metabolism of the microorganisms.

### 4.9. Statistical Analysis

Antimicrobial activity tests were performed in triplicate; one-way unstacked ANOVA by the Tukey test with a confidence level of 95% in MINITAB 16 was applied to determine if a significant difference exists between different kinds of Lf to inhibit growth bacteria.

## 5. Conclusions

We successfully expressed high levels of functional rbLf in *Pichia pastoris* with antimicrobial properties; our experience supports the hypothesis that structural differences in Lf and its derived peptides exert antimicrobial properties. Furthermore, these advances will allow us to study the molecular mechanisms of molecule adhesion and the immune cooperation of Lf against microorganisms with clinical importance.

## Abbreviations

Lf	Lactoferrin
rbLf	recombinant bovine lactoferrin
rbLfcin	recombinant bovine lactoferricin
cbLf	commercial bovine lactoferrin
cbLfcin	commercial bovine lactoferricin
Kan	Kanamycin
